# Editorial: Five grand challenges in toxicology

**DOI:** 10.3389/ftox.2024.1533238

**Published:** 2025-01-21

**Authors:** Bengt Fadeel, Jan Alexander, Sara C. Antunes, Kim Dalhoff, Ellen Fritsche, Helena T. Hogberg, François Huaux, Stina Oredsson, Antonio Pietroiusti, Terje Svingen, Martin F. Wilks

**Affiliations:** ^1^ Institute of Environmental Medicine, Karolinska Institutet, Stockholm, Sweden; ^2^ Norwegian Institute of Public Health, Oslo, Norway; ^3^ Department of Biology, Faculty of Sciences, University of Porto, and Interdisciplinary Center for Marine and Environmental Research, University of Porto, Porto, Portugal; ^4^ Department of Clinical Pharmacology, Bispebjerg Hospital, Copenhagen, Denmark; ^5^ Swiss Centre for Applied Human Toxicology, Basel, Switzerland; ^6^ Department of Pharmaceutical Sciences, University of Basel, Basel, Switzerland; ^7^ Division of Translational Toxicology, National Toxicology Program Interagency Center for the Evaluation of Alternative Toxicological Methods, Durham, NC, United States; ^8^ Université Catholique de Louvain, Louvain-la-Neuve, Belgium; ^9^ Department of Biology, Lund University, Lund, Sweden; ^10^ Saint Camillus International University of Health and Medical Sciences, Rome, Italy; ^11^ National Food Institute, Technical University of Denmark, Kongens Lyngby, Denmark

**Keywords:** challenges, exposome, omics, predictive toxicology, risk assessment

## Introduction

The well-known dictum “the dose makes the poison” is familiar to every student of toxicology, though it is important to add that there is no risk of adverse effects without exposure. This essential point is sometimes overlooked when new and emerging risks such as engineered nanomaterials are being discussed (Fadeel, 2019). The question of the dose of a poisonous substance or toxicant relates to the question of toxicology as a scientific discipline–is toxicology the science of poisons, or has toxicology evolved into a “science of safety”? (Collins et al., 2008). Furthermore, are we ready to shed the predominantly observational science of the past and usher in a new predictive toxicological science of the future? Fifteen years ago, Thomas Hartung lamented that toxicological studies search for rare events using imperfect models–usually addressing one substance at a time (Hartung, 2009). He then listed scientific and strategic developments that are required to implement a new regulatory toxicology including the need for standardization and validation of new approaches, as well as the systematic integration of these approaches into testing strategies (Hartung, 2009). The question is, what should serve as the benchmark given that conventional validation processes often rely on animal tests that may lack adequate validation themselves (Nel and Malloy, 2017). Notwithstanding, it is evident from these discussions that toxicology is both a basic and an applied science. The objectives are thus two-fold: to uncover mechanisms of adverse effects of substances on biological systems while also leveraging this knowledge to protect human health and the environment. To facilitate this translation of knowledge into practice, the flow of information should be bidirectional. Indeed, to promote the field, a dialogue between researchers and regulators is required to identify the kind of knowledge that is needed.

## Grand challenges

The authors of this editorial are editors of the different sections of *Frontiers in Toxicology*, a journal that was launched 5 years ago (in 2019). *Frontiers in Toxicology* is a multidisciplinary journal committed to presenting the latest research on the adverse effects of substances, particles, and mixtures on living organisms and ecosystems, from molecular mechanisms to population effects. *Frontiers in Toxicology* received its first impact factor in 2024. However, we believe that the true impact of the journal lies in providing a forum for the exchange of knowledge in the toxicological community. Here, we address five grand challenges, with the aim of stimulating further discussion.

### Challenge 1: investigation–towards a mechanistic toxicology

Toxicology is sometimes viewed as a descriptive science tasked with cataloguing the effects of substances or drug candidates. However, toxicology as a basic science devoted to the understanding of mechanisms of toxicity covers the spectrum of adverse effects of chemicals, which in turn may unearth fundamental biological mechanisms. Thus, toxicology is not an afterthought. For instance, mechanistic toxicological studies have delivered critical insights in biology and toxicology including an improved understanding of the role of the gut microbiome in xenobiotic metabolism (Koppel et al., 2017), as well as the role of xenobiotic receptors or environmental sensors such as the aryl hydrocarbon receptor (AhR) in health and disease (Suzuki et al., 2020). Furthermore, so-called investigative (or mechanistic) toxicology is gaining momentum in the pharmaceutical sciences (Pognan et al., 2023). Some of the “game-changing” technologies in investigative toxicology, according to a recent survey conducted among medium-sized to large pharmaceutical companies, are organ-on-a-chip microphysiological systems, genomic profiling, and high-content imaging-based assays while the perception of other technologies including the use of stem cells had barely changed when compared to a previous survey (Pognan et al., 2023). It should be emphasized, however, that the ultimate goal of toxicological investigations differs in the pharmaceutical and public health disciplines. In the first case, the goal is to minimize the adverse effects of the drug so that the balance between beneficial and adverse effects is in favor of its use (which implies that some degree of toxicity is considered acceptable), whereas in the second instance, it is obvious that society does not tolerate any toxicity. Indeed, the challenge in occupational medicine is to identify the highest level of exposure at which no adverse effect is elicited. Nevertheless, important lessons can be learned from the pharmaceutical sciences. Computational approaches are being leveraged in drug discovery (Jorgensen, 2004), and physics-driven (quantum mechanics) models as well as data-driven (artificial intelligence) based approaches have been gaining a footing in toxicology too (Kostal, 2023).

### Challenge 2: integration–a problem of multiscale comparisons

Biological pathways are usually defined as a series of interactions among molecules that lead to a certain product or a change in a cell (though it is noted that there are also extracellular biological pathways). Pathway “thinking,” i.e., the view that the adverse effects of substances may be best understood in terms of their disruption of biological (or molecular) pathways, has come to the fore in contemporary toxicology, and it is explicit in the development and use of adverse outcome pathways (AOPs). Indeed, it has been suggested in a recent perspective that the basic tenet of toxicology be recontextualized as “the dose disrupts the pathway” in order to better guide and understand the results of mechanistic toxicological investigations (Suvorov, 2024). The challenge is thus to design experiments that allow for the investigation of pathways as opposed to reductionist studies of one gene or one protein at a time while also making sure that the model systems are relevant for our understanding of adverse effects in humans. In addition, simultaneous exposure to several chemicals is common, and it is not currently clear if the overall level of toxicity is affected in this situation (for instance, it remains to be understood whether exposure to a mixture in which the individual components are present at a concentration below their no-effect-level may induce toxic effects as a result of so-called “dose addition” wherein each component acts on the same target). Considerable advances have been made in recent years with respect to organ-on-a-chip models including a living, breathing microphysiological lung mimic (Huh et al., 2010). Using this bio-inspired device, the authors demonstrated that cyclic mechanical strain accentuated inflammatory responses of the lung to silica nanoparticles. The model is promising though it is noted that a human adenocarcinoma cell line, NCl-H441, was used to impersonate real lung epithelial cells. The use of stem cells (see below) instead of cell lines could potentially bridge the gap between *in vitro* models and the human situation. Moreover, while microphysiological systems are amenable to high-throughput screening (Huh et al., 2010), the additional cost and complexity could hamper their use.

This brings us to one of the major challenges in toxicological testing, namely, how to bridge the divide between *in vitro* models and the *in vivo* situation (which is not to say that human *in vitro* models need to mimic animal models; instead, they should be predictive of relevant biological outcomes in humans which is, after all, the main goal). Furthermore, the impacts of substances play out across multiple spatial and temporal scales, yet we tend to test substances using static model systems which may or may not replicate the biological pathways that come into play in a living organism. Computational approaches can be used to address this problem (Brooks et al., 2020). The challenge is to integrate a multiscale approach wherein biological effects occur at multiple levels of biological organization with input from toxicokinetic and toxicodynamic modeling to better understand what happens when a substance or toxicant is introduced into the body.

The devil is in the details. In toxicology, human cell culture techniques have been adopted for a variety of purposes, including the reduction or replacement of animal experiments. However, animal-derived products are often used meaning that these methods are not completely animal-free. For instance, fetal bovine serum (FBS), which is used to supplement cell culture medium, not only gives rise to animal welfare concerns, but also raises questions related to batch-to-batch variation and the ill-defined nature of FBS, not to mention that species differences may call into question the relevance of the results obtained using standard cell culture medium. However, there is a push towards fully humanized cell culture protocols using chemically defined media (Rafnsdóttir et al., 2023).

### Challenge 3: computation–beware of drowning in a sea of data

The term “omics” in toxicology implies technologies such as genomics, transcriptomics, proteomics, metabolomics, and epigenomics coupled with conventional toxicological assays to investigate the underlying molecular mechanisms of action of toxicants. Sydney Brenner famously complained that molecular biology had become, in his words, “low input, high throughput, no output science,” and it is true that omics-based approaches sometimes are equated with hypothesis-free research, but this is a misguided view as is the suggestion that lists of genes or proteins or metabolites can shed light on biological mechanisms. The methodologies listed above are merely tools, albeit very sophisticated ones, and computational deconvolution is needed to make sense of the sea of data. Hence, omics-based approaches will not necessarily reveal a (novel) toxicological mechanism–the results also need to be anchored in a firm understanding of the biology in question. On the other hand, omics-based approaches yield a global view of the perturbations triggered by a substance or mixture of substances and could thus overcome reductionist tendencies in toxicology. It is important to note that the portfolio of omics techniques is rapidly expanding, and single-cell transcriptomics and proteomics has greatly increased the granularity of toxicological investigations while the recent introduction of spatially resolved transcriptomics heralds a new era in the analysis of gene expression at the tissue level (Meier et al., 2024). The torrential increase in the amount of data generated requires new computational approaches (Canzler et al., 2020), and a new breed of toxicologists well versed in such methods.

Predictive toxicology can be viewed, fundamentally, as the prediction of biological activity from chemical structure; this is also known as structure-activity relationship modeling (Winkler et al., 2013). To this end, the data have to be useful, i.e., of good quality, as well as usable (meaning that other investigators must also be able to access the data). However, predictive toxicology may also refer to the prediction of outcomes in complex (living) systems from observations in simple ones (Svingen, 2022). The challenge is to ensure that *in vitro* models are predictive of *in vivo* outcomes, and that such models can be used to address the impact of substances (toxicants) quantitatively as well as qualitatively. If these challenges are met, this could significantly overhaul human health risk assessment (Svingen, 2022). The advent of artificial intelligence (AI) has further expanded the toolbox of toxicologists. Thus, machine learning approaches could promote predictive toxicology and could perhaps be used to support risk assessment and decision making (Lin and Chou, 2022). Machine learning gives computers the ability to learn without explicitly being programmed. However, “learning” may be too anthropomorphic; instead, AI algorithms enable pattern recognition which could guide (human) toxicologists and risk assessors. Understanding the reliability of AI-based tools in toxicology is, indeed, a challenge. Machine learning also paves the way from a deterministic towards a probabilistic risk assessment (see below). Probabilistic risk assessment and Bayesian analysis (named after the English statistician Thomas Bayes whose posthumous paper “An Essay Towards Solving a Problem in the Doctrine of Chances” forms the basis of what is now called Bayes’ theorem) are methodologies incorporating uncertainties and variability that provide estimates of the range and likelihood of a hazard or risk in the assessment (US Environmental Protection Agency, 2014).

### Challenge 4: regulation–rise of new approach methodologies

Historically, hazard assessment has been based mainly on animal data, which does not necessarily provide a mechanistic understanding of the observed effects. However, considerable attempts have been made in regulatory toxicology to transit from *in vivo* (animal) studies to mechanism-based, human-relevant *in vitro* studies (Nel and Malloy, 2017). The US Environmental Protection Agency (EPA) initiated the ToxCast, or “toxicity forecaster”, program to develop decision support systems based on *in vitro* screening results to aid in the prioritization of environmental chemicals for further investigation (Kleinstreuer et al., 2014). Indeed, conventional cell cultures serve as a useful screening tool. However, to better mimic the complexity of a living organism, multicellular organotypic models are being developed (Fritsche et al., 2021). Here, advances in stem cell biology, especially the development of the human induced pluripotent stem cell (iPSC) technology, have provided a significant boost. Developmental neurotoxicology with its battery of stem cell and primary cell based new approach methodologies (NAMs) can be viewed as a test pilot for assessing complex endpoints (Fritsche et al., 2021). The challenge is to implement these new technologies in a regulatory setting. Making predictions regarding human health risks based on cell models is non-trivial. However, AOPs provide a conceptual framework for the organization of toxicological knowledge, from molecular-level perturbations of a biological system to adverse outcomes at the level of biological organization of regulatory relevance (Ankley et al., 2010). Toxicogenomics data can be used to enrich AOPs (Saarimäki et al., 2023). It is important, however, to distinguish between an adverse response and an adaptive response as the distinction between the two is critical to regulatory decision making (Keller et al., 2012).

Overall, testing approaches that are reliable and relevant for human biology are needed, and NAMs are being developed with this in mind. This challenge is addressed, for instance, in the Partnership for the Assessment of Risks from Chemicals (PARC), a multinational project aligned with the European Union’s Chemicals Strategy for Sustainability and the European Green Deal (www.eu-parc.eu). Similarly, the NIH Common Fund’s Complement Animal Research in Experimentation (Complement-ARIE) program is aimed at speeding up the development, standardization, validation, and use of NAMs (www.commonfund.nih.gov). NAMs are defined as any technology or methodology that can provide information on chemical hazard and risk assessment without the use of animals. However, NAMs are not necessarily newly developed methods; instead, it is their implementation in regulatory decision making that may be considered new (Stucki et al., 2022). Thus, regulatory acceptance is key, and a dialogue with regulatory agencies is needed. Ultimately, integration of NAMs into chemical risk assessment practices will depend not only on demonstrating their scientific merits, but also on addressing perspectives of risk assessors and the public on how to assess risk and the associated uncertainties (Bearth et al., 2024).

The translation of the results of *in vitro* or animal studies to humans is further complicated by factors, which may not be systematically considered in such studies, but which may strongly affect the final biological effect. For instance, pre-existing diseases may cause an increased susceptibility to the effects of chemicals. Co- and cumulative exposures also need to be considered, as well as the sex and life stage (age) of the exposed individual (Varshavsky et al., 2023). Moreover, certain minority groups may display an unpredictable response to chemicals. Indeed, sex-divergent immune responses were disclosed in a recent study of individuals undergoing gender affirming hormone treatment (James and Brodin, 2024). Traditional risk assessment has used default uncertainty factors to address inter-individual and inter-species differences, but new probabilistic methods and Bayesian approaches promise to support risk assessment to better protect all populations at risk (Maertens et al., 2024).

Ecological risk assessment is a stepwise evaluation approach wherein assessment parameters at the different stages rely on sensitive toxicological assays. The major challenge has been the identification and validation of biomarkers capable of reporting issues before they manifest in effects at the individual or population level, thus allowing decision makers to act and implement measures to safeguard ecosystems. In this context, concerted efforts are being made to identify the gaps in the current mammalian-centric high-throughput screening assay landscape with respect to ecologically relevant endpoints (Villeneuve et al., 2019). Human health and environmental risk assessments are often performed in isolation, and a key challenge is therefore to implement an integrated risk assessment in which the different aspects of risk assessment are consolidated into a “one health” paradigm (Saarimäki et al., 2023). This is also emphasized in the safe-and-sustainable-by-design (SSbD) and circular economy approach or vision which guides the innovation process towards creating chemicals and materials that are more sustainable and safer for humans and the environment (Kümmerer et al., 2020).

### Challenge 5: education–fostering the future toxicologists

Paracelsus, the Father of Toxicology and one of the first physicians to recognize the role of chemistry in medicine, believed that only those who practiced an art knew it: “the patients are your textbook.” He sought the kind of knowledge that cannot be achieved solely through scholastic disputations, and therefore embarked on a series of extensive travels around Europe including spending a year as a physician and university professor in Basel where he gave lectures in medicine based on his own observations, not on ancient tracts written centuries ago. This episode reminds us that toxicology thrives on new knowledge and new approaches, and it illustrates how observation (data collection) enables toxicologists to challenge prevailing dogma. The practice of critical thinking should be instilled, first and foremost, in our students who are the toxicologists of the future. It is equally important to convey to young scientists interested in toxicology that toxicology is a full-fledged scientific discipline with a uniquely interdisciplinary nature. Hence, toxicology integrates knowledge from a wide range of fields, including biology, chemistry, pharmacology, medicine, epidemiology, statistics, mathematics, computer science, and so on. Due to this interdisciplinarity, toxicology is usually taught in various departments, thus providing toxicologists with a broad and versatile education. However, interdisciplinary education also requires ample time for synthesis and reflection. Furthermore, toxicology relies on the use of advanced tools and instruments, and an appreciation for analytical chemistry (including mass spectrometry), high-throughput biology, and computer science and statistics (big data, large cohort studies) is required to enable the next-generation of toxicologists to tackle the many challenges of contemporary toxicology, as exemplified here.

## Closing remarks

*Minority Report* (2002) is a science fiction movie in which Tom Cruise portrays the chief officer of a “precrime” unit that stops crimes before they take place. However, toxicologists are not clairvoyants who are able to foresee and prevent impending disaster. Instead, the predictive toxicology paradigm refers to the use of models and methods (including computational methods) complementary to or as a replacement of classical descriptive toxicology; this also implies a shift away from the conventional “one-substance-at-a-time” science towards a holistic science that addresses real-life exposures throughout the lifespan of an individual (aka the exposome) (Barouki et al., 2022). The “exposome” has been regarded by some experts as a nebulous concept. However, the exposome may be viewed as a bridge between epidemiological and toxicological studies. Indeed, a stronger focus on the totality of environmental exposures could galvanize efforts to unearth exposure-effect relationships at the individual and population level.

Another important branch of toxicology involves the assessment of the effects of xenobiotics on ecosystems and the biota (the animal and plant life) that they contain. Predictive toxicology can provide an early warning of more complex environmental problems. Furthermore, the conjunction of rising contaminant levels in the environment and climate change makes it essential to understand and anticipate issues that are more difficult to resolve and require time-consuming and costly restoration measures (Noyes et al., 2009).

Thus, one of the overarching themes during the past 15 years or more has been the transformation of toxicology from a predominantly observational (empirical) science to a predominantly predictive science that would take toxicity pathways into account (Krewski et al., 2010). Computational approaches are integral to this new predictive toxicology, as discussed here. However, it is prudent to consider the lessons of the past as we look to the future (European Environment Agency, 2001).

**FIGURE 1:**
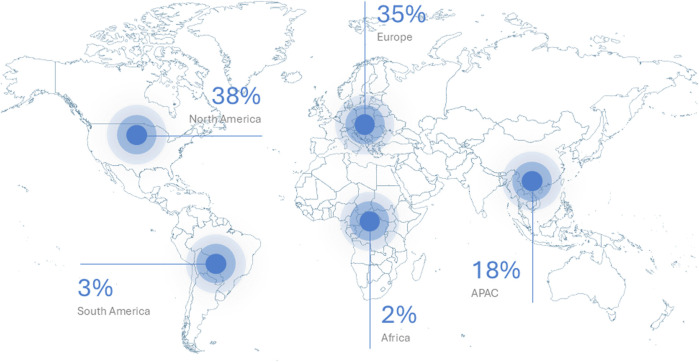
*Frontiers in Toxicology* provides a forum for the worldwide community of toxicologists. The figure shows the geographic distribution of all articles to date (June 2024) based on submitting author country. Source: *Frontiers of Toxicology* Editorial Office.

In this article, we have presented five grand challenges in an attempt to frame contemporary toxicology, and we hope that this brief overview will stimulate further discussion. *Frontiers in Toxicology* provides a forum for the worldwide community of toxicologists (Figure 1). The first article (editorial) published in the journal focused on engineered nanomaterials (Fadeel, 2019), while the most cited article to date addresses conventional and innovative techniques for the detection of micro- and nanoplastics (Mariano et al., 2021), a topic of great concern. The second and third most cited articles in *Frontiers in Toxicology* provide an overview of the use of new approach methodologies (NAMs) to meet regulatory requirements (Stucki et al., 2022) and a survey of the reproductive impacts of PFAS (per- and polyfluorinated alkyl substances, or “forever chemicals”) (Chambers et al., 2021), respectively. *Frontiers in Toxicology* currently has close to 700 editorial board members from more than 50 countries, most of which are located in Europe, North America, and the Asia-Pacific (APAC). However, we also welcome editorial board members and authors from other regions. Together, we hope to advance the field of toxicology, bridging fundamental scientific discoveries and their practical applications to promote human health.

## Author’s note

The authors are Specialty Chief Editors of the different sections of Frontiers in Toxicology and BF is also Field Chief Editor of the journal.
